# Influence of the Biliary System on Biliary Bacteria Revealed by Bacterial Communities of the Human Biliary and Upper Digestive Tracts

**DOI:** 10.1371/journal.pone.0150519

**Published:** 2016-03-01

**Authors:** Fuqiang Ye, Hongzhang Shen, Zhen Li, Fei Meng, Lei Li, Jianfeng Yang, Ying Chen, Xiaochen Bo, Xiaofeng Zhang, Ming Ni

**Affiliations:** 1 Department of Biotechnology, Beijing Institute of Radiation Medicine, Beijing, People’s Republic of China; 2 Department of Gastroenterology, Hangzhou First People’s Hospital, Hangzhou, Zhejiang, People’s Republic of China; 3 Department of Research Service, Zhiyuan Inspection Medical Institute, Hangzhou, Zhejiang, People’s Republic of China; 4 Department of Radiation Toxicology and Oncology, Beijing Institute of Radiation Medicine, Beijing, People’s Republic of China; National Institute for Viral Disease Control and Prevention, CDC, China, CHINA

## Abstract

Biliary bacteria have been implicated in gallstone pathogenesis, though a clear understanding of their composition and source is lacking. Moreover, the effects of the biliary environment, which is known to be generally hostile to most bacteria, on biliary bacteria are unclear. Here, we investigated the bacterial communities of the biliary tract, duodenum, stomach, and oral cavity from six gallstone patients by using 16S rRNA amplicon sequencing. We found that all observed biliary bacteria were detectable in the upper digestive tract. The biliary microbiota had a comparatively higher similarity with the duodenal microbiota, versus those of the other regions, but with a reduced diversity. Although the majority of identified bacteria were greatly diminished in bile samples, three *Enterobacteriaceae* genera (*Escherichia*, *Klebsiella*, and an unclassified genus) and *Pyramidobacter* were abundant in bile. Predictive functional analysis indicated enhanced abilities of environmental information processing and cell motility of biliary bacteria. Our study provides evidence for the potential source of biliary bacteria, and illustrates the influence of the biliary system on biliary bacterial communities.

## Introduction

The biliary microbiota is considered to be a vital factor in gallstone pathogenesis [1–5]. Many bacteria, such as *Escherichia coli*, *Klebsiella pneumoniae* and *Enterococcus faecium*, have been identified through cultivation [6–8] or polymerase chain reaction in bile or gallstone samples previously [9, 10]. Recently, the application of next-generation sequencing on biliary samples provided a more comprehensive understanding of biliary bacterial community and expanded the microbiota detected in the human biliary tract [11]. In our previous work, by using both whole-metagenome shotgun sequencing (WMS) and bacterial 16S rRNA amplicon sequencing (referred to as ‘16S sequencing’) to study bile samples from gallstone patients, we observed great bacterial community heterogeneity among patients, and identified 13 novel biliary bacterial species such as *Prevotella pallens*, *Streptococcus infantis*, and *Porphyromonas endodontalis* [12].

However, another important issue about where biliary bacteria come from still remains unclear. Generally, retrograde infection of gut bacteria from the duodenum is considered to be the likely primary source of biliary infection [13, 14]. Besides, bacterial invasion through the portal venous system also contributes to biliary infection with relatively low morbidity [15, 16]. Duodenum anatomically connects with the biliary tract via the sphincter of Oddi, and the sphincter of Oddi seems to be the only way which must be passed for possible ascending infection of gut bacteria. However, to our knowledge, there has not yet been a comparative study of the biliary versus the duodenal microbiota. Previous study has shown that the microbiota of three upper digestive tract sites (represented as saliva, stomach biopsy and duodenum biopsy samples) have a commonality of detected bacterial taxa [17]. Human upper digestive tract samples, including saliva, gastric fluid, and duodenal mucus, were also found to differ greatly in their bacterial composition from fecal samples [18, 19]. Thus, considering the smaller anatomical distance to the biliary tract, the upper digestive tract rather than the distal gut is more likely to be the primary source of biliary bacteria.

With the protection of the sphincter of Oddi from enteric bacterial invasion, the antimicrobial activity of bile salts and the immunological defense system [8], the biliary microenvironment is believed to be generally hostile to most bacteria. Thus, it is of great interest to use the human digestive tract microbiota as a reference to investigate the likely effect of the biliary microenvironment on biliary bacteria, and the extent to which bacterial composition is altered in the microbe-inhospitable biliary tract. Moreover, due to the great microbial heterogeneity among individuals, such comparative study should be performed within the same individuals. Till now, there has been no such investigations.

In this study, we compared the bacterial communities of the biliary and upper digestive tracts of six gallstone patients. We aim to obtain evidence for the source of biliary bacteria and to observe how the biliary microbiota may be affected by its surrounding environment.

## Materials and Methods

### Sample collection

All patients were diagnosed with choledocholithiasis (mean age: 69.3 ± 14.6 years; 4 men, 2 women) by using B-mode ultrasonography and computed tomography. Each individual had gallstones detected in the common bile duct. No one had occurrences of gallbladder gallstones. These patients did not receive antibiotics for at least three months before their endoscopic retrograde cholangiopancreatography (ERCP) procedure. All patients provided written informed consent upon enrollment. The study conformed to the ethical guidelines of the 1975 Declaration of Helsinki and was approved by the Institutional Review Board of Hangzhou First People’s Hospital. All samples were collected at Hangzhou First People’s Hospital. Salivary samples were collected after the patients gargled with 20 mL of sterile saline water. Patients expectorated their mouthwash into sterile sputum cups. The gastric fluid, duodenal fluid, and bile samples were collected by using strictly sterile side-viewing endoscopes (TJF240/JF-260V; Olympus Optical, Tokyo, Japan). Measures were taken to avoid sampling artifacts or cross contamination among samples in the same patient. During advancement of the endoscope from the mouth into the stomach and into the duodenum, the work channel of the endoscope kept itself clean and uncontaminated by avoiding any pumping action. Gastric fluid, duodenal fluid, and bile samples (2–5 mL of each sample type) were aspirated into sterile sputum cups through sterile catheters which passed through the work channel. Each catheter was used only once and was replaced with a new catheter prior to collecting the next sample. If gastric fluid was invisible in the stomach, sterile saline water would be injected through the catheter and gastric flushing fluid was collected. When the endoscope reached the duodenum, a new sterile catheter was used to inject sterile saline water and aspirate the duodenal flushing fluid. Sterile sphincterotome catheters (V-SYSTEM; KD-V411M-0725; Olympus Optical) were used to suck out bile samples (2–5 mL) from the common bile duct. All samples were stored at -80°C until further processing.

### DNA extraction

Total DNA was extracted from each noncentrifuged sample with the Invitrogen Purelink Genomic DNA Mini Kit (Life Technologies, Carlsbad, CA, USA) following manufacturer’s instruction. A Qubit 2.0 Fluorometer (Life Technologies) was used to quantify all the DNA, and the E-Gel electrophoresis system (Life Technologies) was used to examine the DNA quality.

### 16S rRNA amplicon sequencing

The V3–V4 region of the bacterial 16S rRNA gene was amplified by using universal primer pairs 356F (5’CCTACGGGNGGCWGCAG3’) and 803R (5’GACTACHVGGGTATCTAATCC3’). Detailed protocols for the two-step 16S rRNA gene amplification and library construction procedures are reported elsewhere [12, 20]. In the first-round PCR, 16S primers and overhang adapters compatible with the Illumina Nextera XT kit (Illumina, San Diego, CA, USA) were employed to amplify the V3-V4 region of 16S rRNA. In the second-round PCR, Illumina adapters and sample barcodes were added to the purified PCR products from the first step. Three tubes of sterile water were used as negative controls during the whole amplification and library preparation process. The negative control samples had no detectable DNA products when evaluated by the E-gel electrophoresis system (Life Technologies). The DNA libraries were sequenced on the Illumina MiSeq platform to generate 2 × 250-bp paired-end reads.

### Taxonomic profiles and pathway profiles of 16S sequencing data

FLASH v1.2.11 [21] merged pair-end reads into ~460-bp sequences. QIIME v1.8.0 [22] were employed to analyse the merged reads. Quality control was performed by using the script “split_libraries_fastq.py” in QIIME with following parameters “-n 0 -p 0.75 -q 19 -r 3”. Chimeras were removed by aligning reads to sequences in the Greengenes Database (version Aug, 2013) with USEARCH v6.1 [23]. Remaining reads were clustered into operational taxonomic units (OTUs) at the 97% similarity level by UCLUST v1.2.22 [23]. Each representative OTU was assigned a taxonomic rank with Ribosomal Database Project classifier v2.2 [24] at a confidence level of 0.85. Phylogenetic Investigation of Communities by Reconstruction of Unobserved States (PICRUSt) [25] analysis was performed to generate Kyoto Encyclopedia of Genes and Genomes (KEGG) pathway profiles as previously described [26]. In brief, the OTUs used for PICRUSt analysis were picked by using the QIIME script “pick_closed_reference_otus.py” at the 97% similarity level. Taking the generated OTU table as input, PICRUSt would output an annotated table of predicted metabolic functions at KEGG pathway level by using the scripts “normalize_by_copy_number.py” and “predict_metagenomes.py”. The pathway profiles were employed for further comparison.

### Statistical analysis

To avoid bias introduced by varied sequencing depth of these samples, diversity indices (observed OTU and Bray-Crutis dissimilarity) were calculated by randomly re-sampling the reads of each sample to a uniform number for 1,000 times. Mean diversity indices were used for downstream analysis. Analysis of similarity (ANOSIM) using Bray-Crutis dissimilarity was performed by using the software Past (http://folk.uio.no/ohammer/past/). Pearson correlation coefficients between the microbiota of the biliary tract and those of each upper digestive tract site were calculated by using R (v3.1.2). In order to detect differentially abundant genera and KEGG pathways in the biliary tract relative to other sampling sites, Wilcoxon rank-sum test (R project, v3.1.2) was employed to compare bile samples and the upper digestive tract samples. Differences with *P* < 0.05 were considered to be statistically significant.

### Deposition of sequence data

All sequence data were deposited in the National Center for Biotechnology Information (NCBI) under BioProject PRJNA290974. All samples were registered in NCBI under BioSample numbers SAMN03938337–SAMN03938359.

## Results

### All biliary bacteria were detectable in the upper digestive tract

By using 16S sequencing, we obtained an average of 12,299 high-quality reads per sample (range: 3,088–83,985, the gastric fluid sample from P6 was excluded for further analysis due to its ultra-low sequencing depth), and detected a total of 1,398 operational taxonomic units (OTUs) (S1 Table). Among the bile samples, *Proteobacteria*, *Firmicutes*, *Bacteroidetes*, *Actinobacteria*, *Fusobacteria*, *Synergistetes* and *TM7* were identified at the phylum level, the first five of which are common inhabitants of the human digestive tract [17, 27]. Six of these seven bacterial phyla have been found in biliary samples in previous studies, with the exception being *Synergistetes* [7, 11, 28–31].

**Fig 1:**
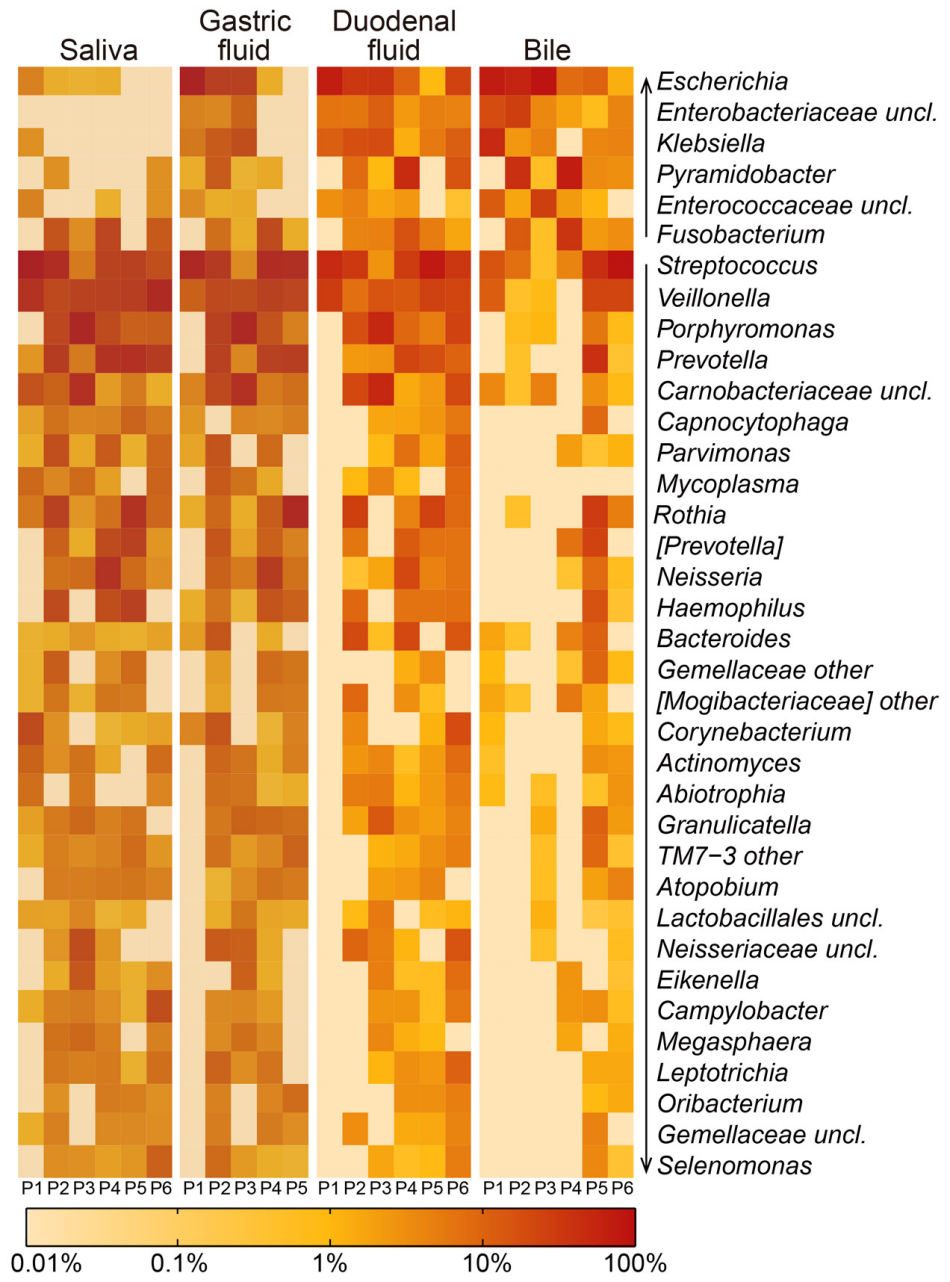
Distribution of genera that were highly prevalent across the four body sites. Only genera with ≥0.01% abundance in at least 12 samples are presented. OTUs that could not be assigned at the genus level were labelled as “unclassified” (uncl.). Genera that were poorly defined in Greengenes database were labelled as “other”. *[Prevotella]* and *[Mogibacteriaceae]* are provisional taxonomical assignments of operational taxonomic units by Greengenes. The upward and downward arrows indicated genera whose abundance was increased and decreased in bile samples, relative to the other regions, respectively.

We found that all biliary bacteria were detectable in the upper digestive tract. These seven aforementioned biliary phyla were all identified at one or more digestive tract sites from the same patients (S2 Table). At the genus level, 53 bacterial genera were found in bile samples with ≥0.1% abundance, all of which were included within the 102 genera (≥0.1% abundance) identified in at least one site of the upper digestive tract (S3 Table). Among them, *Streptococcus*, *Veillonella*, *Prevotella* and *Rothia* were most prevalent in these patients with ≥2% average relative abundances at each body site (Fig 1, S3 Table).

**Fig 2:**
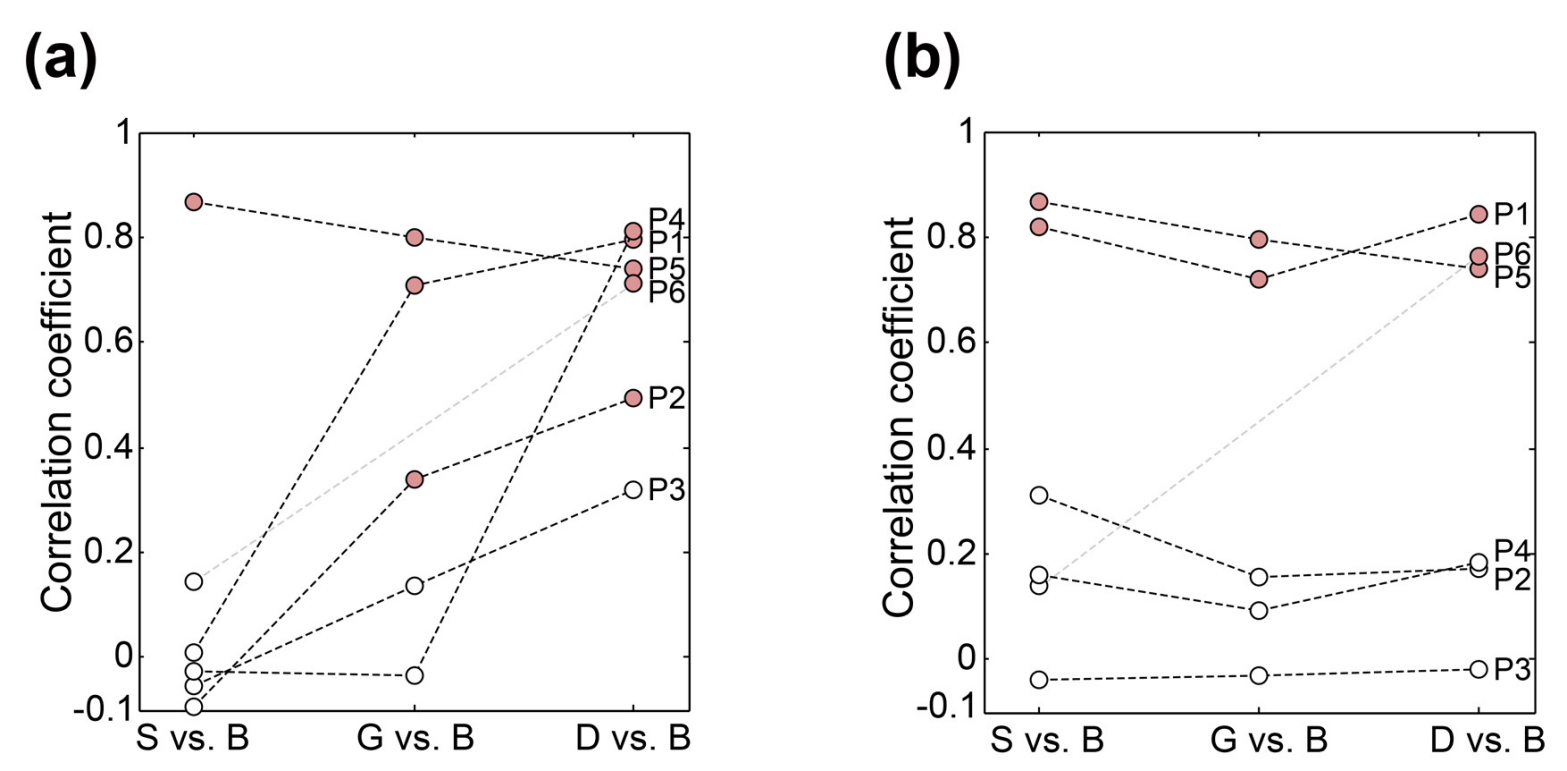
Correlation coefficients between the microbiota of each upper digestive tract site and the biliary microbiota. For each patient, correlation coefficients between the microbiota of the biliary tract and other sampling sites were calculated when *Pyramidobacter* and three *Enterobacteriaceae* genera (*Escherichia*, *Klebsiella*, and an unclassified genus) in bile samples were included (a) and excluded (b). Saliva, gastric fluid, duodenal fluid, and bile are denoted as “S”, “G”, “D”, and “B”, respectively, on the x-axis. Correlations that were statistically significant are indicated with red circles.

Next, we investigated the correlation between the microbiota of each upper digestive tract site and the biliary microbiota. Generally, the biliary microbiota were more similar to those of the duodenal fluid samples than to those of the gastric fluid or saliva samples (Fig 2A). In five patients (P1–P4, and P6), the bacterial composition of the bile samples correlated increasingly well with, in order, the bacterial composition of the same patient’s saliva, gastric fluid, and duodenal fluid. In one exceptional patient, P5, all upper digestive tract sites had a high similarity of bacterial composition with the bile sample (Pearson correlation coefficient > 0.73, *P* < 2.6 × 10^−7^). The dominant biliary genera in P5 (e.g. *Prevotella*, *Rothia*, and *Haemophilus*) were distinct from those of other patients, and were relatively more aligned with the patient’s salivary microbiota (S3 Table). The observation in P5 revealed variability of bacterial composition among these patients.

*Pyramidobacter* and three *Enterobacteriaceae* genera (*Escherichia*, *Klebsiella*, and an unclassified genus) were highly abundant in the majority of bile samples. To further trace the potential source of the rare biliary microbes, we re-calculated the correlation coefficients between bile and other sites after excluding these four genera (Fig 2B). Interestingly, reminiscent of our observations with P5, P1’s bile also had a bacterial distribution that was highly similar to that observed in his saliva, gastric fluid, and duodenal fluid (Pearson correlation coefficients > 0.72, *P* < 3.4 × 10^−6^). This result suggests that the salivary microbiota may also contribute to the biliary microbiota, though the overlap may be overshadowed in most cases by the relative dominance of duodenal genera. Following the aforementioned exclusion, the significant correlation between the duodenal and the biliary microbiota was maintained in P6 but not in P2 or P4.

### Diverse changes of the biliary microbiota relative to the upper digestive tract microbiota

Diversity differences in the biliary microbiota relative to the upper digestive tract microbiota were observed. Alpha diversity indices indicated that bile samples had a reduced bacterial diversity than the upper digestive tract samples. To avoid potential bias of varied sequencing depths, we randomly re-sampled the reads of these samples to a uniform number 3,088 which was the smallest read number among these samples, and calculated microbial diversity indices. The alpha diversity of bile samples, measured by observed OTUs, was significantly smaller in bile samples than in samples from the other three upper digestive tract sites (Wilcoxon rank-sum test, bile *vs*. saliva, *P* = 0.015; bile *vs*. gastric fluid, *P* = 0.03; bile *vs*. duodenal fluid, *P* = 0.041). Analysis of similarity (ANOSIM) using Bray-Crutis dissimilarity demonstrated significant differences between bile and saliva samples (*P* = 0.003), but not between bile and gastric or duodenal fluid samples (bile *vs*. gastric fluid, *P* = 0.125; bile *vs*. duodenal fluid, *P* = 0.3772).

Although genera that occurred in bile were also detected in the upper digestive tract, the relative abundance and prevalence of these bacteria differed greatly. Of the 36 prevalent genera identified in at least 12/23 samples, 30 (83.3%) had a lower average abundance in bile than in one or more other sites, and 6 (16.7%) had a higher average abundance in bile (Fig 1). With respect to bacterial prevalence among these patients, 17 biliary genera were present in only 1–2 patients’ bile samples, though these same 17 genera were observed in the duodenal fluid in 3–5 patients (S3 Table).

**Fig 3:**
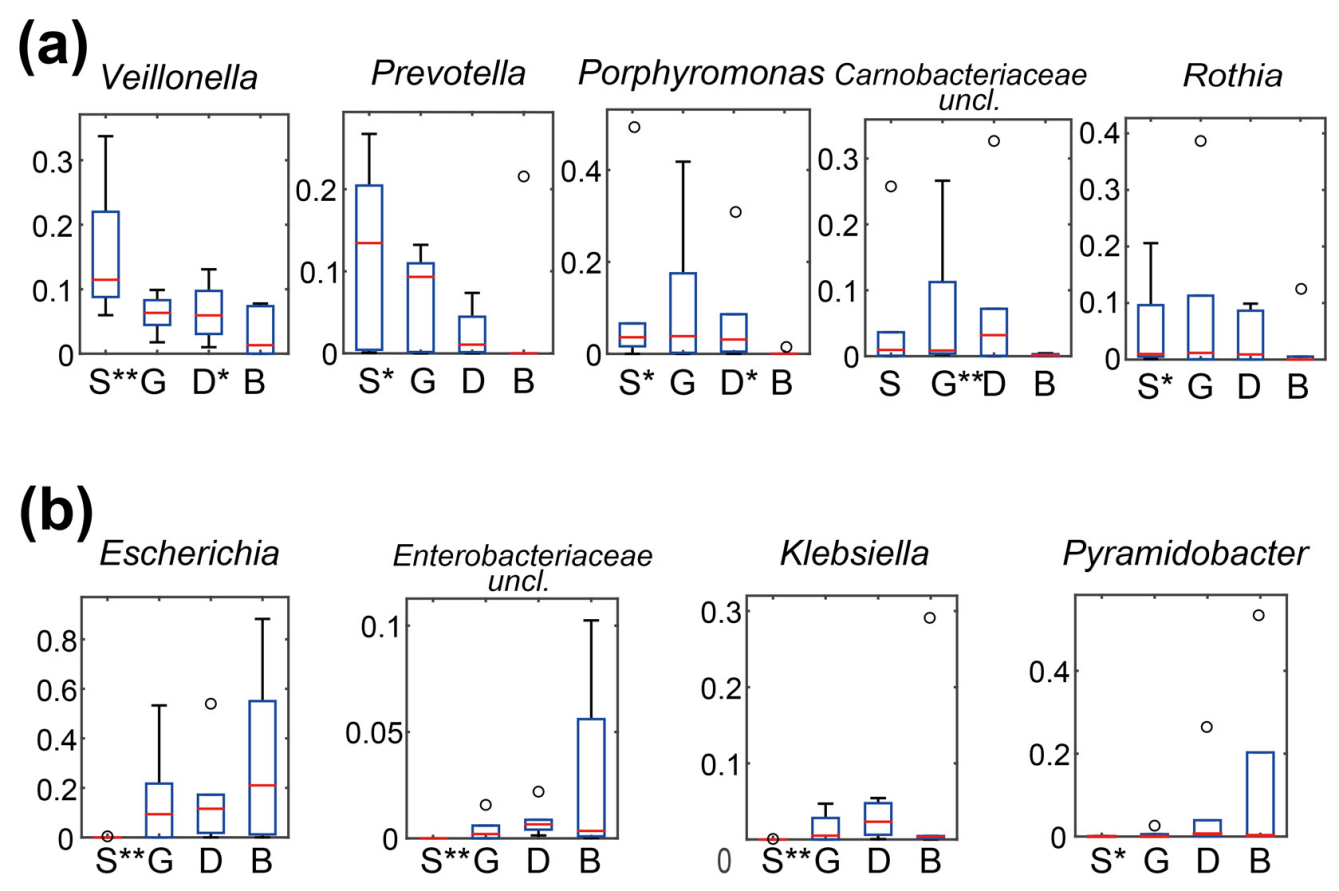
Distribution of differentially abundant genera among all sampling sites. Genera were filtered by the criteria of with a ≥10% abundance in at least one sampling site and with a ≥0.01% abundance in at least 12 samples. Then, filtered genera that were decreased (a) and increased (b) in relative abundance in bile samples compared to at least one of the other body sites were illustrated. The y-axis represents relative abundance. Body sites that showed statistically significant differences relative to bile samples are labelled with an “*”. Boxes represent the 25th- to 75th-percentile interquartile range. The red lines inside the boxes indicate the median values, and the whiskers represent the most extreme values within 1.5 times of the interquartile range. Red lines for genus *Pyramidobacter* have been especially thickened for a better visualization as the median values were close to zero. Outlier values are represented as circles. **P < 0.01, *P < 0.05.

Moreover, it is notable that the relative abundances of some taxa exhibited an increasing or decreasing tendency from saliva through gastric fluid and duodenal fluid to bile. Bacterial genera whose relative abundances were significantly different between bile and one or more upper digestive tract sites are illustrated in Fig 3A and 3B. For *Veillonella* and *Prevotella*, we observed a gradually decreasing tendency from saliva to bile (Fig 3A). *Prevotella* was undetectable in bile for most patients. Meanwhile, the overall abundances of *Porphyromonas*, *Rothia*, and an unclassified *Carnobacteriaceae* genus were maintained at somewhat comparable levels in the upper digestive tract sites, with a sudden and drastic decrease in bile. These five genera that were found to be decreased in bile are often identified in the human oral cavity, stomach, and duodenum [17, 27], implying that the biliary environment might impose higher survival and selective pressures upon these bacteria.

Four genera had significantly increased abundance in bile (Fig 3B). Among them, three genera, namely *Escherichia*, *Klebsiella*, and an unclassified genus, belong to the *Enterobacteriaceae* family, and have been isolated from bile cultures previously [6–8, 11]. Their increased abundance in bile might be associated with a bile-resistant ability [3]. The fourth genus, *Pyramidobacter*, which belongs to the phylum *Synergistetes*, was highly enriched in P2 and P4. *Pyramidobacter* has been isolated mainly from the human oral cavity [32, 33] and also, albeit less often, from the human small intestine [34]. It was also identified from human bile samples in our previous study [12]. The archetype species of this genus, *P*. *piscolens*, encodes a multidrug transporter protein AcrB that is related to bacterial bile resistance [3]; if this gene is shared with other *Pyramidobacter* species, it may explain, at least in part, the enrichment of *Pyramidobacter* in bile.

### Biliary bacteria had differentially abundant inferred pathways

**Fig 4:**
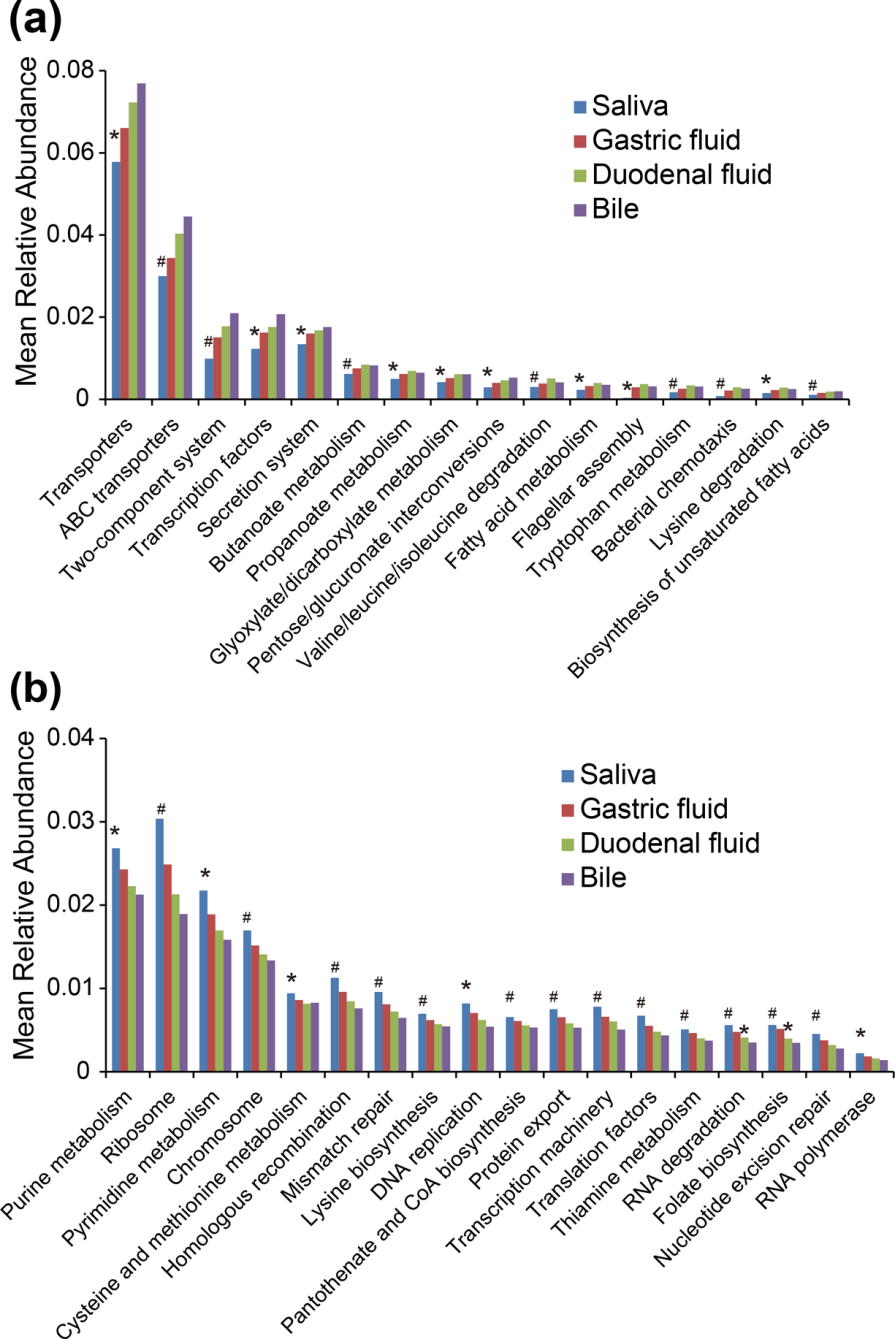
Bacterial metabolic pathways of saliva, gastric fluid, duodenal fluid, and bile samples. KEGG pathways with higher (a) and lower (b) abundance in bile samples, relative to the other sites, were illustrated. Body sites with significant differences relative to bile samples are labelled with an “*” (*P* < 0.05) or “#” (*P* < 0.01) above the bars.

We constructed the predictive functional profiles of the bacterial communities of the four sampling sites by Phylogenetic Investigation of Communities by Reconstruction of Unobserved States (PICRUSt) [25], and determined that biliary bacteria had differentially abundant inferred Kyoto Encyclopedia of Genes and Genomes (KEGG) pathways when compared to the upper digestive tract microbiota (Fig 4A and 4B). We identified 24 significantly enriched pathways and 41 depleted pathways (all *P* < 0.05) in bile samples (S4 Table).

The enriched pathways in bile samples were related to environmental information processing, cell motility, carbohydrate metabolism, lipid metabolism, and amino acid metabolism (Fig 4A). Environmental information processing pathways, such as ABC transporters, two-component system, and secretion system, were dominant in these samples. We observed an increasing tendency of their relative abundances from saliva to bile. Considering the high level of antimicrobial bile salts and immunoglobulins in the biliary tract, this result suggests that bacteria that can survive in bile may have a greater ability to respond to environmental information than bacteria in other sampling sites. Bacterial chemotaxis and flagellar assembly pathways were also enriched. These cell motility-related functions enable bacteria to move toward favorable living conditions [35], and thus might play important roles in their survival and colonization within the biliary tract. With respect to energy source and nutrition utilization, carbohydrate metabolic pathways (e.g. butanoate, propanoate, glyoxylate, and dicarboxylate metabolism), lipid metabolic pathways (e.g. fatty acid metabolism and biosynthesis of unsaturated fatty acids), and amino acid metabolic pathways (e.g. tryptophan metabolism and degradation of valine, leucine, isoleucine, and lysine) were also well represented in biliary bacteria.

The pathways with relatively low representation in the biliary microbiota were involved in genetic information processing, nucleotide metabolism, amino acid metabolism, and metabolism of cofactors and vitamins (Fig 4B). Genetic information processing pathways included DNA replication, transcription machinery, mismatch repair, RNA degradation, RNA polymerase, homologous recombination, and protein export. Purine metabolism and pyrimidine metabolism were related to nucleotide metabolism. Metabolism of cofactors and vitamins involved folate biosynthesis, pantothenate/CoA biosynthesis, and thiamine metabolism. Amino acid metabolism related pathways cysteine/methionine metabolism and lysine biosynthesis had decreased abundances in bile samples. Moreover, the biliary microbiota had a relatively higher abundance of degradation processes (e.g. lysine degradation) combined with a relatively low representation of biosynthesis processes (e.g. lysine biosynthesis). Taken together, the differences among pathways involved in carbohydrate, lipid, amino acid, nucleotide, and cofactor/vitamin metabolism suggest that biliary bacteria may have different energy and nutrient sources from the upper digestive tract microbiota.

## Discussion

Our results support the hypothesis that biliary bacteria originate from retrograde infection of gut bacteria. All observed biliary bacteria could be identified in the upper digestive tract with 16S sequencing, with the biliary microbiota being relatively more similar to the duodenal microbiota. Furthermore, we observed the influence of the biliary environment on biliary bacterial composition, as revealed by diverse alterations of the biliary microbiota relative to the upper digestive tract microbiota. The changes might be attributed to the anatomical structure and liquid features of the biliary tree. The sphincter of Oddi, through which bile from the biliary tract unidirectionally flows into the duodenum, acts as an anatomical barrier and protects the biliary system from enteric bacterial invasion [8]. Moreover, the antimicrobial activity of bile salts and the immunoglobulins in the biliary tract also exert selective pressures on biliary bacteria [16].

The difference in bacterial relative abundance in bile samples might imply the difference in their bile-resistant ability. *Escherichia* and *Klebsiella* both had increased abundance in bile samples relative to the other sampling sites. Species from both genera have been cultured from human bile samples, such as *E*. *coli*, *K*. *pneumonia* and *K*. *oxytoca* [6–8, 11]. Other bacterial taxa, such as *Veillonella* and *Prevotella*, had reduced abundances in the biliary tract when compared to the upper digestive tract, and they are less frequently cultured from biliary samples. Several genes have been found to be potentially related to bile resistance, including *bsh*, *acrA*, *acrB*, *emrA*, *emrB*, and *tolC*. By searching the KEGG GENOME database, we found that the genomes of species belonging to those aforementioned abundant *Escherichia* and *Klebsiella* genera harbored more bile-resistant genes than those of *Veillonella*, *Prevotella*, *Rothia*, and *Porphyromonas*. One bacterial genera of interest is *Pyramidobacter*, which belongs to the phylum *Synergistetes*. It was highly abundant in some bile samples when compared to the corresponding upper digestive tract samples. Species from *Pyramidobacter* has been isolated from the human oral cavity [32, 33] and small intestine [34], and we detected this genera in human bile samples in our previous study for the first time [12]. AcrB, a multidrug efflux pump protein related to bile resistance, was encoded by the archetype species of this genera, *P*. *piscolens*, which may explain, to some extent, the high abundance of *Pyramidobacter* in the biliary tree. Improvement of cultivation-based methods would assist to the identification of *Pyramidobacter* in biliary samples, and bile-resistance mechanism employed by this novel but poorly defined bacterial genus also needs further investigation.

Predictive functional profiles of all sampling sites, assessed by PICRUSt, determined that biliary bacteria might have increased abilities of environmental information processing and cell motility, which are crucial functions for bacterial life. Differential representation of pathways related to energy and nutrient sources also suggest that there may be an influence of the biliary system on resident bacterial function. Propanoate metabolism and ABC transporters were also found to be enriched in bile samples by using whole-metagenome shotgun sequencing in our previous study [12]. It should be noted that most of the differentially abundant pathways were detected between saliva and bile samples (Fig 4A and 4B), which was consistent with the comparisons based on microbial distributions (Figs 2 and 3). Bacterial microbial communities which have similar microbial structures tend to have similar functions (including pathways). As the biliary microbiota resembled increasingly well, in order, those of the saliva, the gastric fluid, and the duodenal fluid samples, the biliary samples might have fewer statistically differential pathways relative to the duodenal fluid and the gastric fluid samples than the saliva samples. In addition to 16S sequencing, more advanced technologies, such as whole-metagenome shotgun sequencing, metatranscriptomic, metametabolomic and metaproteomic technologies, will contribute to a more comprehensive picture of biliary bacterial function and the microbe-host interaction.

Taken together, our results provide evidence for the human duodenum as the primary source of biliary bacteria, and characterizes the influence of the biliary system on the biliary microbiota. The aim of our study is to investigate how bacterial microbiota vary in the human biliary and upper digestive tracts. With the 23 samples from 6 patients, we performed comparisons among microbial communities from each sampling site and observed obvious variation trends of the similarities between the biliary microbiota and those in other upper digestive tract sites. However, it is likely that there are other microbial variation patterns we did not find due to the limited sample size. With respect to the microbial community, the biliary tract is a poorly explored microenvironment compared to other human body sites. More studies, especially those about the role of microbes in gallstone pathogenesis, are needed to understand the biliary microbial ecology in the future.

## Supporting Information

S1 Table Statistical analysis of 16S sequencing results. (XLSX)

S2 Table Relative abundances of bacterial phyla present in samples from each region. (XLSX)

S3 Table Relative abundances of bacterial genera present in samples from each region. Only genera with ≥0.1% abundance from at least one sample are presented here.
(XLSX)

S4 Table Differential KEGG pathways represented in the biliary tract microbiota versus those represented in the upper digestive tract microbiota. (XLSX)
